# Tonsillectomy and social media: An investigative analysis of educational tonsillectomy content on TikTok

**DOI:** 10.1002/hsr2.618

**Published:** 2022-06-27

**Authors:** Jithin John, Rohun Gupta, Pushtee Jhaveri, Eduardo M. Leon, Eric Cox, Jonathan Raskin, Neil J. Khatter, Ricky Sayal, Adam Folbe

**Affiliations:** ^1^ School of Medicine Oakland University William Beaumont School of Medicine Rochester Michigan USA; ^2^ Department of Arts and Sciences University of Georgia Athens Georgia USA; ^3^ Department of Otolaryngology Beaumont Health Systems Royal Oak Michigan USA; ^4^ ENT Specialists, P.C. Novi Michigan USA

**Keywords:** otolaryngology, social media, TikTok, tonsillectomy, video platform

## 1 INTRODUCTION

The use of social media platforms has aided in spreading information more rapidly than ever before. More recently, emerging as the fastest growing social media platform and one of the fastest to gain popularity is TikTok.^1^ As of November 15, 2021, TikTok is the most downloaded free application on both Apple and Google Play Store. TikTok is a video‐sharing platform that creators use to post about endless topics through various forms such as dances, songs, or dialogs. Anyone can become a creator on TikTok, and this provides the opportunity for people to both views and add videos about trending topics. The app generates premade templates and audio for users to use, making it easy for anyone to create content. For viewers, the videos are intentionally designed to be short, allowing users to watch more content in a reduced amount of time. Furthermore, TikTok trends seem to be the most viral videos as it showcases current trends, icons, and lifestyles. Aside from entertainment, educational health content and personal experiences of different health conditions are also frequently posted.^2^ Since most of the viewership on TikTok videos is randomly selected rather than only followers/friends, like most other popular social media platforms, information can spread faster than ever before.^1^, ^2^

For the medical community, this creates a platform for providers and patients to share information anywhere from patient experiences to medical conditions and their treatment. Studies have already been conducted on this for topics, such as concussions, cosmetic procedures, and acne.^2^, ^3^, ^4^ Given the popularity and rapid ability of TikTok to circulate information, we were interested in analyzing otolaryngology content. Prior studies have been conducted analyzing otolaryngology content on other social media platforms, such as Youtube and Twitter; however, our study is the first to explore and characterize otolaryngology content and specifically tonsillectomy content on TikTok.^5^

Tonsillectomy is the surgical removal of the tonsils to treat commonly recurrent tonsillitis and hypertrophy of the tonsils causing respiratory problems, and less commonly for peritonsillar abscess, biopsy, and as an access to other structures.^6^, ^7^ Studies indicate that tonsillectomy in patients with recurrent tonsilitis provides improved quality of life and decreased upper respiratory tract infections and sinus infections.^7^ The most common complications of tonsillectomy include postoperative hemorrhage and infections, which may require immediate surgical interventions.^6^, ^7^, ^8^ On the basis of the most recent published data, tonsillectomies are among the most common surgical procedures in the United States, with 289,000 ambulatory procedures done annually in children less than 15 years of age.^8^ With tonsillectomy being the most commonly performed otolaryngology procedure, analyzing this topic will provide a plethora of informational content.^7^ The purpose of this study is to analyze content related to otolaryngology, specifically in relation to tonsillectomy, found on TikTok. We aim to recommend methods to improve patient education, patient–provider interactions, and shared decision‐making.

## 2 MATERIALS AND METHODS

​​This is a cross‐sectional study that utilizes TikTok, a social media platform utilized for creating, sharing, and discovering short video clips from users. Utilizing TikTok's search feature, we looked for videos that utilized the words “tonsillectomy.” Our inclusion criteria were any videos related to tonsillectomy and otolaryngology and our exclusion criteria included non‐English videos, private accounts, duplicate videos, and unrelated videos. Videos that met the exclusion criteria were removed (*n* = 12). The remaining videos (*n* = 188) were analyzed and characterized using the DISCERN questionnaire by two independent reviewers (Pushtee Jhaveri and Jonathan Raskin). The DISCERN questionnaire is a 16‐question survey used to assess the quality of published treatment information reliably.^8^ DISCERN has shown to be a reliable instrument to judge the quality of health information.^9^ If the average score differed by greater than 3 points, an additional reviewer (Jithin John) was utilized to maintain accurate results.

## 3 STATISTICAL ANALYSIS

The DISCERN scores were recorded for each video as numerical values (0–5) for each of the DISCERN questionnaires. The average score of each video was collected from the reviewers, which was then averaged to create a collective average score for each video. DISCERN scores were recorded in Microsoft Excel (Redmon, Washington), and tests for means, standard deviations, and significance levels were calculated utilizing the standard statistical formulas. The *p*‐values were calculated using a one‐tailed *T*‐test. *p*‐values were considered statistically significant if *p* < 0.05.

## 4 RESULTS

**Figure 1:**
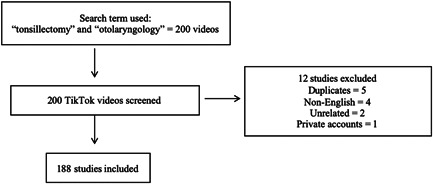
Flow sheet demonstrating how videos were selected

**Figure 2:**
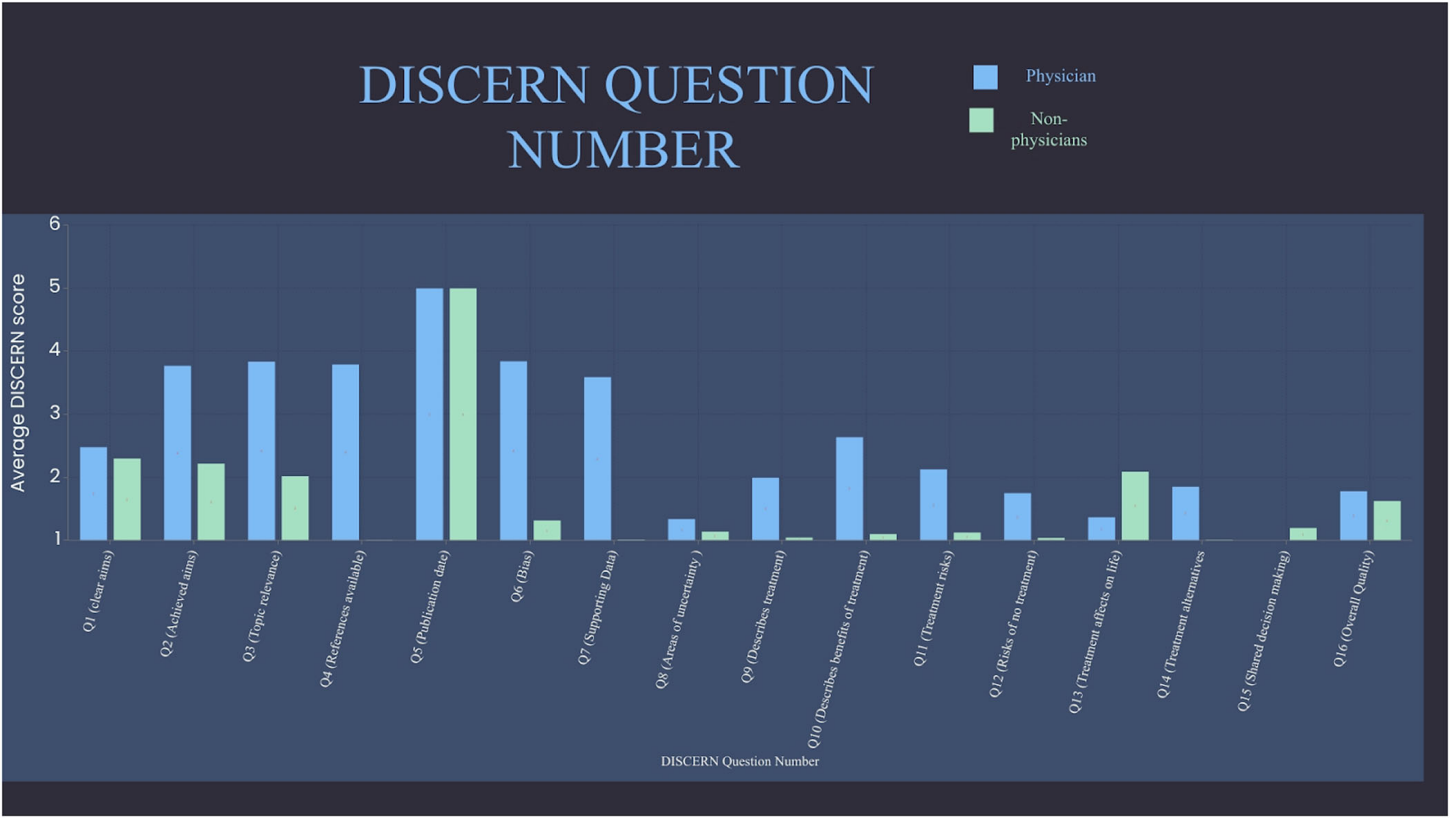
Average DISCERN scores per question comparing physicians and nonphysicians

**Figure 3:**
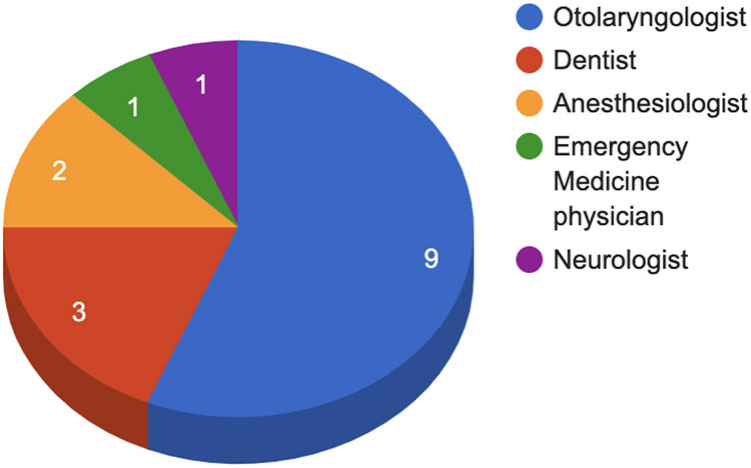
User type of physicians posting “Tonsillectomy” video content on TikTok

From our search strategy, the top 200 videos, of which 188 videos met our inclusion and exclusion criteria. The study included 188 total videos for analysis (Figure 1). The videos had a total combined 6,248,294 likes and 82,875 comments and were shared 91,737 times. The average DISCERN score for all of the videos combined was 2.24. The collected DISCERN scores had a range from 0 to 4, with a standard deviation of 0.54. Since TikTok is a video‐sharing platform used to watch videos that are less than a few minutes long, some of the questions from the DISCERN questionnaire were not applicable. Some of these questions included: support for decision making, referring to areas of uncertainty, and use of references, as evidenced by the low score in both physician and nonphysician group videos (Figure 2). Of the total 188 videos, 16 were created by physicians, 6 were animations, and 166 were personal experience videos by nonphysicians. Out of the 16 physician‐created videos, 9 were otolaryngologists, while the rest of the 7 included dentists (3), anesthesiologists (2), emergency medicine physicians (1), and a neurologist (1) (Figure 3). ​​When stratified, the DISCERN score was higher for videos created by physicians (DISCERN average 2.57) than for videos created by nonphysicians (DISCERN average 1.65) (*p* < 0.001) (Table 1). The number of male and female otolaryngologists and nonotolaryngologists videos was also collected and characterized, as shown in (Table 2).

**Table 1:** Overview of TikTok videos related to tonsillectomy

	Number of videos	Mean number of likes	Mean number of comments	Mean number of shares	Mean DISCERN score
User type					
Physician	16	92,512.26	1472.60	2170.87	2.57
Nonphysicians individuals	166	16,546.97	236.89	3759.33	1.65
Animations	6	349,544.33	3537.67	219.27	2.5
Physician specialty					
Otolaryngology	9	90,684.00	18,501.00	2757.00	2.42
Nonotolaryngologist physicians	7	82,001.26	806.86	1118.43	2.81
Video type					
Educational	21	157,856.00	2156.30	2731.55	2.44
Personal story	167	16,619.53	236.25	219.05	1.643
Gender					
Male	29	63,206.61	797.5	1285.25	1.93
Female	153	15,491.13	256.60	216.83	1.67
Other	0	0	0	0	0

**Table 2:** Characterization of physician content creators according to gender

Physician content creators (*n *= 16)	Number of males and females
Otolaryngologist (*n* = 9)	Male: 7
	Female: 2
Nonotolaryngologist (*n* = 7)	Male: 4
	Female: 3

## 5 DISCUSSION

The purpose of this study is to characterize TikTok videos related to otolaryngology, specifically related to tonsillectomy. Through this characterization, the goal was to propose an approach to improve patient education, patient–provider interactions, and shared‐decision making. There have been published analyses of otolaryngology content on other social media platforms, such as Facebook, Youtube, etc. To our knowledge, this is the only study to analyze and characterize both tonsillectomy content and any otolaryngology content on TikTok. Therefore, there were no comparable studies in relation to the characterization of any otolaryngology content.

Tonsillectomy is one of the most commonly performed otolaryngology surgery, therefore we believe this search result (“Tonsillectomy”) serves as a representative sample of the available otolaryngology content on TikTok. The findings in this study suggest that otolaryngologists have an opportunity to pioneer novel content and educate the general public. Since animations (average DISCERN score: 2.5) had a similar score to physician‐made videos (average DISCERN score: 2.57), informational videos can be made through animations. Although, it is understandable that physicians may not have the expertise to make animated video content. We also suggest content creators improve explanations of areas of uncertainty, risks of not having treatment, and treatment effects on life, as shown by the low DISCERN score (Figure 2). Lastly, it is also recommended that videos use styles/themes that are currently in trend, as this may lead to higher viewership, as noted during our search. The DISCERN scores of physician‐made videos had a statistically significant (*p* < 0.001) higher score than nonphysician videos, and higher likes and comments, using this information, we believe that by engaging patients on social media platforms, otolaryngologists can increase public awareness and education in regard to common conditions and procedures performed by otolaryngologists, thereby leading to improved satisfaction and long‐term outcomes.

This study has some limitations to note. Since TikTok is a rapidly growing platform, search results vary daily. This is due to the confidential search algorithm TikTok uses and is likely based on current trends. DISCERN questionnaire, the questionnaire used in this study, is a popular tool used to analyze written publications. However, we believe this tool may not be the best instrument to judge the quality of videos. Another scoring method needs to be developed and validated to rate the quality of educational videos because the DISCERN questionnaire was initially created to score written publications only, and no other validated method is available to score videos. We encourage otolaryngologists to study current social media trends to better understand patient perspectives, thereby increasing public education and leading to improved patient outcomes.

## AUTHOR CONTRIBUTIONS

Jithin John, Rohun Gupta, Pushtee Jhaveri, Jonathan Raskin, and Neil J. Khatter devised the project and directed planning. Jithin John, Rohun Gupta, Pushtee Jhaveri, Jonathan Raskin, Neil J. Khatter, and Eric Cox analyzed the data. Jithin John, Rohun Gupta, Eric Cox, Ricky Sayal, and Adam Folbe wrote the manuscript with input from all authors.

## TRANSPARENCY STATEMENT

The lead author (manuscript guarantor) affirms that this manuscript is an honest, accurate, and transparent account of the study being reported; that no important aspects of the study have been omitted; and that any discrepancies from the study as planned (and, if relevant, registered) have been explained.

## CONFLICTS OF INTEREST

The authors declare no conflicts of interest.
